# An investigation into the reliability of a mobile app designed to assess orthodontic treatment need and severity

**DOI:** 10.1038/s41415-022-4246-2

**Published:** 2022-05-27

**Authors:** Sukbir Nandra, Nicola Crawford, Daniel Burford, Nikolaos Pandis, Martyn T. Cobourne, Jadbinder Seehra

**Affiliations:** 41415106840001 Department of Orthodontics, Medway Maritime Hospital, Windmill Road, Gillingham, Kent, ME7 5NY, UK; 41415106840002 Department of Orthodontics and Dentofacial Orthopedics, Dental School/Medical Faculty, University of Bern, Freiburgstrasse7 CH-3010, Bern, Switzerland; 41415106840003 Department of Orthodontics, Faculty of Dentistry, Oral and Craniofacial Sciences, King´s College London, Floor 25, Guy´s Hospital, Guy´s and St Thomas´ NHS Foundation Trust, London, SE1 9RT, UK

## Abstract

**Aim** To investigate reliability of the Easy IOTN app between clinicians with different levels of experience in determining Index of Orthodontic Treatment Need (IOTN) Dental Health Component (DHC) and Aesthetic Component (AC) scores from study models. The accuracy of each clinician in discriminating treatment need using the app against the 'gold standard' conventional assessment at the threshold of treatment acceptance criteria was also explored.

**Materials and methods** In total, 150 sets of pre-treatment study models were assessed by six clinicians using the app on two separate occasions (T1 and T2). A single IOTN-calibrated clinician also scored the models using the conventional technique. Clinician scores for both intra- and inter-rater reliability were assessed using Cohen's Kappa. The performance of each clinician in discriminating treatment need using the app against the conventional assessment method at the threshold of treatment acceptance criteria was also assessed using the area under the curve-receiver operating characteristic.

**Results** The intra-rater agreement for the clinician undertaking the conventional assessment of the models was 1.0. Intra-rater agreement scores for clinicians using the Easy IOTN app ranged between 0.37-0.87 (DHC) and 0.22-0.44 (AC). Inter-rater agreement scores at T2 were 0.59 (DHC) and 0.23 (AC). Based on the IOTN DHC, all clinicians displayed an excellent level of accuracy in determining malocclusions qualifying for treatment (range 81.7-90.0%). Based on the IOTN AC, all clinicians showed an acceptable level of accuracy in determining malocclusions qualifying for treatment (range 71.9-79.2%).

**Conclusions** The Easy IOTN app was shown to have moderate inter-rater reliability. Variation in the intra-rater reliability was evident between clinicians of different grades/level of experience. Importantly, the diagnostic accuracy of the app to discriminate between malocclusions that qualify for NHS treatment was rated as excellent (IOTN DHC) and acceptable (IOTN AC) and independent of clinician grade or level of experience.

## Key points

- The Easy IOTN app was developed to assist in scoring a malocclusion according to the Index of Orthodontic Treatment Need, which categorises malocclusions using a Dental Health Component and Aesthetic Component.
- The study findings have shown that the Easy IOTN app has moderate inter-rater reliability.
- The diagnostic accuracy of the app in discriminating between malocclusions that qualify for NHS treatment was rated as excellent (IOTN DHC) and acceptable (IOTN AC).
- The Easy IOTN app can be used by non-specialists to assess treatment need and severity.

## Introduction

According to the World Health Organisation, a healthcare index should be reproducible, valid, simple, sensitive throughout the scale and have the ability to be performed quickly.^1^ Indices of orthodontic treatment need, such as the Index of Orthodontic Treatment Need (IOTN),^2^ are employed to plan the provision of orthodontic treatment for populations so that those deemed to need treatment can 'qualify' and receive it. This index categorises malocclusions using two separate components: the Dental Health Component (DHC) and Aesthetic Component (AC). Currently, the threshold of acceptance for NHS orthodontic treatment in England and Wales is IOTN DHC 3, AC 6.

In the past two decades, the use of mobile devices such as smartphones and tablets has increased exponentially. Applications (apps) are small, specialised programs that can be downloaded onto a mobile device. Due to their transportability, ability to update and speed and ease of use they have become an ideal tool for quick reference and entertainment. The use of mobile applications in healthcare has also grown. A survey in 2015 found that 54% of the Royal College of Physicians membership stated they use apps to support their clinical work, with 42% of members believing these apps were essential or very important to their daily work.^3^ Healthcare apps are aimed at either clinicians or patients. However, the information provided in many apps is not regulated and often is not evidence-based. Consequently, apps may encourage patients to make inappropriate decisions relating to their healthcare.^4^

Orthodontic apps aimed at clinicians are focused on clinical education, treatment planning, diagnostic aids and product marketing.^4^^,^^5^^,^^6^ The Easy IOTN app aims to assist in improving the standard and accuracy of orthodontic referrals, as well as supporting the training of junior dentists.^7^ This freely downloaded app uses a step-by-step process which recognises the key orthodontic features to indicate the IOTN score for a particular malocclusion. For DHC scoring, the user is requested to input specific clinical features of the malocclusion. The app then leads the user onto the AC scoring section and requests a front-facing photograph of the patient's teeth. This clinical photograph is then compared to ten colour photographs displaying increasing aesthetic detriment to rank the patient's level of dental attractiveness. The final page of the app provides the DHC and AC scores for the patient and makes suggestions on whether the patient may or may not qualify for NHS orthodontic treatment. Combining healthcare tools with technology is an innovative way of improving clinical effectiveness; however, this new technology should be validated and proven to be reliable. Within the literature, limited studies have been undertaken assessing the reliability of orthodontic diagnostic apps.^8^ In contrast, within the medical field, conflicting data exist regarding the diagnostic app reliability.^9^^,^^10^

The primary aim of this study was to investigate reliability of the Easy IOTN app between clinicians with different levels of experience in determining IOTN DHC and AC scores from study models. Furthermore, the accuracy of each clinician in discriminating treatment need using the Easy IOTN app against the 'gold standard' conventional assessment at the threshold of NHS treatment acceptance criteria was explored.

## Materials and methods

This study was classified as a service evaluation by the Research and Innovation office at Medway Maritime Hospital (MMH) (R&D reference 1143). No patient-identifiable data were collected as part of the study protocol. Complete high-quality standardised pre-treatment study models representing a range of malocclusions were identified from the orthodontic department at MMH. Prior to selecting the models, it was agreed by consensus between the authors that a range of models representing varying degrees of severity as classified by IOTN DHC (1-5) should be included. As the sample of models was being selected from a secondary care environment, it was expected that most of the models would represent malocclusions classified within the severe need of treatment category (IOTN DHC 5 and 4). Study models of patients requiring orthognathic surgery and cleft lip and palate patients were excluded. The selection of these models was undertaken by a member of staff not involved in scoring of the models. All models were anonymised and consecutively numbered. An additional 20 models representing milder malocclusions (IOTN DHC 3-1) were sourced from two local private practices. Consent for the use of these study models was obtained previously. Based on a previous reliability study for an index of orthodontic treatment need,^11^ a sample size of 150 sets of pre-treatment study models was agreed as an adequate sample size.

All orthodontic clinicians working in the orthodontic department at MMH agreed to participate in the study. This included seven clinicians of varying grades and different levels of experience. These were three orthodontic consultants (C1, C2, C3), one specialist orthodontist (SO), one dentist with special interest in orthodontics (DWSI), one postgraduate orthodontic student (PS) and one orthodontic therapist (OT). One senior clinician (C1) calibrated in scoring IOTN was selected to be the 'expert' examiner and scored the study models using the conventional technique.^12^ This involved scoring treatment need directly from study models using an IOTN ruler, conventional ruler (mm), pencil and the associated IOTN DHC descriptors and AC photographs. The remaining six clinicians were assigned to score the models using the Easy IOTN app after undergoing calibration by completing the CPD training section on the app, which consists of 30 questions. All clinicians, excluding the DWSI and OT, regularly used IOTN to score malocclusions on their routine clinics. The DSWI and OT worked on treatment-only clinics, which did not require the regular use of IOTN. Both the DHC and AC of IOTN can be assessed from study models when a dental cast protocol is applied.^13^ This protocol assumes the worst-case scenario with regard to the malocclusion. Based on this protocol, an adapted protocol was devised to ensure consistency during the scoring of the models at both time-points with the app (Table 1).

**Table 1:** Protocol for scoring models with the Easy IOTN app

Situation	Protocol
Missing teeth	If a permanent tooth is absent (not including 7s and 8s): Assume the tooth is impacted if the space remaining for an unerupted tooth is less than or equal to 4 mm If the space is more than 4 mm, assume the tooth is missing (ie the patient has hypodontia)
Overjet 3.5-6 mm	Assume the lips are incompetent
Reverse overjet	If more than 1 mm, assume that masticatory and speech issues are present
Crossbites	Assume no discrepancy between RCP and ICP, ie no mandibular displacement on closure (in the conventional assessment, a displacement of more than 2 mm is assumed - worst case scenario)
Contact point displacements	Only record the worst displacement between permanent teeth (do not record displacements between primary and permanent teeth)
Overbite	If there is labial gingival recession of the lower incisors or palatal stripping of the gingival mucosa of upper incisors, then the overbite is traumatic

Models were scored at two separate time points, (T1 and T2) separated by a four-week interval. The clinicians were requested to provide a DHC and AC score for all 150 study models. All the study models were scored in the same session with no time limitations. Examiners were provided with a flexible plastic ruler (mm) and a data collection sheet to record their IOTN scores. The expert clinician, who scored the models conventionally, was also provided with crib sheets with generic DHC IOTN score descriptions and the AC photographs.

## Statistical analysis

Descriptive statistics were used to summarise the study characteristics. Clinicians' scores for both intra- and inter-rater reliability were assessed using Cohen's Kappa. The performance of each clinician in discriminating treatment need using the Easy IOTN app against the 'gold standard' conventional assessment at the threshold of NHS treatment acceptance criteria (IOTN DHC 3, AC 6) was assessed using the area under the curve-receiver operating characteristics (AUC-ROC) curve. This analysis summarises the overall diagnostic accuracy of a test by considering values from 0-1, where a value of 0 indicates a perfectly inaccurate test and a value of 1 reflects a perfectly accurate test. The following parameters were used to rank the accuracy of each clinician: 50% suggests no discrimination (that is, ability to diagnose patients with and without the disease or condition based on the test), 70-80% is considered acceptable, 80-90% is considered excellent and more than 90% is considered outstanding.^14^All statistical analyses were performed using STATA software version 16.1 (Stata Corporation, College Station, Texas, USA) and R Software version 4.0.3 (R Foundation for Statistical Computing, Vienna, Austria).

## Results

For the participating clinicians, the median number of years since obtaining a primary dental qualification was 20 years (IQR 25) (Table 2). The number of malocclusion models per IOTN category (DHC and AC) included in this study are shown in Table 3.

**Table 2:** Number of years of experience since initial qualification of each participating clinician

Clinician grade	Years qualified
Postgraduate student (PS)	5
Specialist orthodontist (SO)	7
Orthodontic therapist (OT)	7
Consultant 2 (C2)	20
Consultant 3 (C3)	27
Dentist with special interest in orthodontics (DWSI)	32
Consultant 1 (C1)	32

**Table 3:** Number of models in each category of IOTN (DHC and AC) (N = 150)

DHC	Number of models	AC	Number of models
5a	18	10	2
5i	34	9	26
4a	19	8	15
4d	15	7	28
4e	2	6	14
4f	2	5	18
4h	28	4	32
4l	1	3	12
4m	3	2	2
4t	4	1	1
3a	12		
3d	8		
3e	1		
2d	3		

The intra-rater agreement for the senior clinician (C1) undertaking conventional assessment of the models was 1.0 for both IOTN DHC and AC. Intra-rater agreement scores for clinicians using the Easy IOTN app are shown in Table 4. In terms of DHC scores, both consultants (C2 and C3) and the OT demonstrated a good level of agreement. The SO and PS both had a very good level of agreement. The DSWI was the only clinician to have fair agreement in scoring. AC scoring agreement was lower than DHC scoring agreement for all clinicians. There was a fair level of agreement in the scores given by both consultants, the DWSI and the PS. The SO and orthodontic therapist had a moderate level of agreement in scores. Inter-rater agreement of DHC and AC scores given by clinicians using the Easy IOTN app is shown in Table 5. DHC inter-rater agreement improved at T2 in comparison to T1; however, only a moderate level of agreement was evident. Kappa scores for AC scoring at both T1 and T2 both demonstrated fair agreement and remained consistent at both time points.

**Table 4:** Intra-rater reliability Kappa scores for six clinicians who undertook scoring of models using the Easy IOTN app (<0.2 = poor; 0.21-0.40 = fair; 0.41-0.60 = moderate; 0.61-0.80 = good; 0.81-1.00 = very good agreement)

Clinician grade	DHC	AC
Postgraduate student (PS)	0.81	0.40
Specialist orthodontist (SO)	0.87	0.44
Orthodontic therapist (OT)	0.77	0.56
Consultant 2 (C2)	0.64	0.22
Consultant 3 (C3)	0.75	0.39
Dentist with special interest in orthodontics (DWSI)	0.37	0.28

**Table 5:** Inter-rater reliability Kappa scores for six clinicians who undertook scoring of models using the Easy IOTN app (two assessments four weeks apart) (<0.2 = poor; 0.21-0.40 = fair; 0.41-0.60 = moderate; 0.61-0.80 = good; 0.81-1.00 = very good agreement)

IOTN	T1	T2
DHC	0.48	0.59
AC	0.24	0.23

**Fig. 1:**
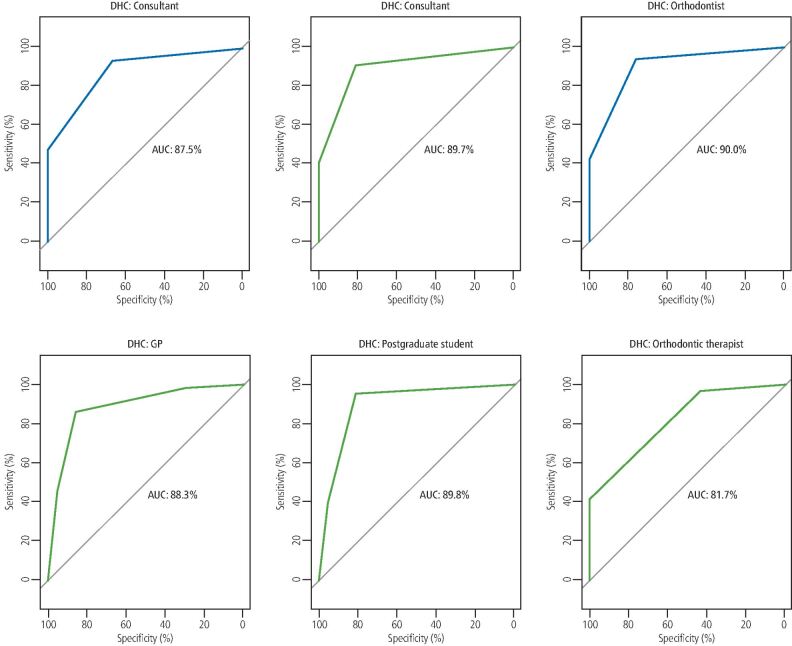
AUC-ROC curves for each clinician depicting the level of accuracy for each clinician (range 81.7-90.0%) in terms of discriminating treatment acceptability/need against the 'gold standard' conventional assessment and at the threshold of treatment acceptance criteria (IOTN DHC)

**Fig. 2:**
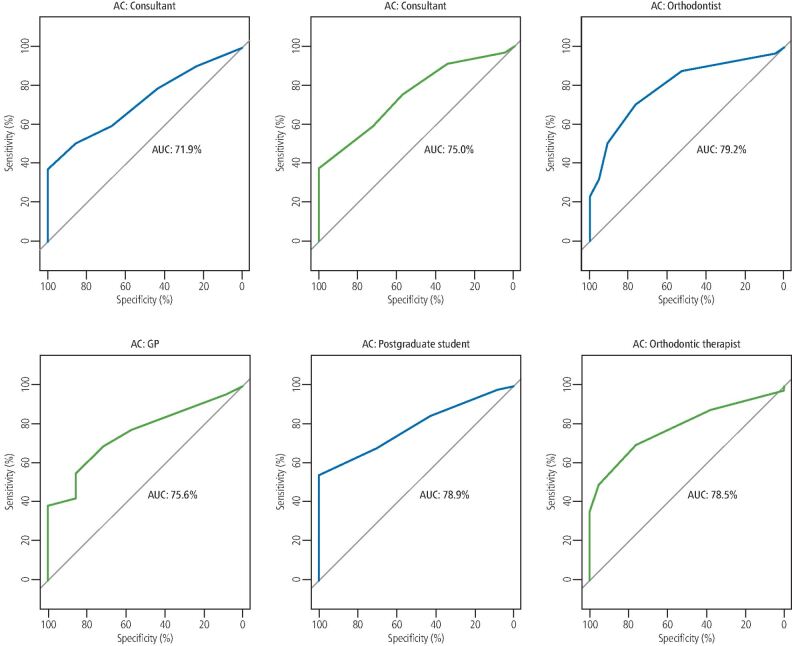
AUC-ROC curves for each clinician depicting the level of accuracy for each clinician (range 71.9-79.2%) in terms of discriminating treatment acceptability/need against the 'gold standard' conventional assessment and at the threshold of treatment acceptance criteria (IOTN AC)

AUC-ROC curves were used to depict the accuracy of each clinician in discriminating treatment need using the Easy IOTN app against the 'gold standard' conventional assessment at the threshold of treatment acceptance criteria (DHC 3, AC 6). Based on DHC, all clinicians displayed an excellent level of accuracy in determining those malocclusions qualifying for treatment (range 81.7-90.0%) (Fig. 1). Based on AC, all clinicians showed an acceptable level of accuracy in determining those malocclusions qualifying for treatment (range 71.9-79.2%) (Fig. 2).

## Discussion

This study has demonstrated that when using the Easy IOTN app to assess orthodontic treatment need from patient study models, a fair to very good intra-rater agreement and moderate level inter-rater agreement for IOTN DHC scores is evident. In contrast, both the intra- and inter-rater agreement for IOTN AC scores was at a fair level of agreement. Importantly, the accuracy of the app to discriminate between malocclusions that qualify for NHS treatment was rated at excellent (IOTN DHC) and acceptable (IOTN AC) and is independent of the clinicians' grade or level of experience.

The app rater scores appear to be comparable with previous investigations of IOTN reliability using the conventional method of scoring. The use and knowledge of IOTN among general dental practitioners (GDPs) in Scotland was found to vary, with only a moderate level of agreement between DHC scores (mean Kappa score of 0.42).^15^ Furthermore, the inter-rater agreement of dental registrants was found to range between fair to good (0.25-0.74).^16^ The results from these studies indicate that the reliability of IOTN DHC scoring using the Easy IOTN app is currently similar to scoring IOTN DHC using the conventional technique. Notably, both intra-rater and inter-rater agreement scores in this study were lower for AC in comparison to DHC. This is not an uncommon observation, as a poor level of agreement between AC scores given by dental registrants has been reported.^16^There is a general opinion that AC scoring is inconsistent because it does not completely reflect certain aesthetic aspects of a malocclusion, such as the degree of overjet, lip competence and spacing.^17^ Consequently, the subjective nature of the AC may have contributed to the lower agreement scores achieved in this study. It was also observed that DHC inter-rater agreement levels improved at T2. This suggests that the more times the examiners in this study used the Easy IOTN app, the more consistent their scoring was among each other. This is consistent with conventional scoring of IOTN DHC scores, which have been found to be reliable over time.^18^

It was also noted that clinicians with less years of dental experience had marginally higher intra-rater reliability scores for both IOTN DHC and AC. The reason for this is not clear but it may be because junior clinicians are more comfortable with using new technology such as apps.^19^ Another potential explanation could be that the junior clinicians underwent their initial training more recently than the others and therefore retain more knowledge. Indeed, there is evidence showing that the teaching of IOTN is successful during undergraduate training^20^ but this knowledge appears to reduce after graduation.^15^

The DWSI notably had the lowest intra-rater agreement scores (IOTN DHC and AC). Those such as DWSI are qualified dentists who choose to work within a specific dental speciality and undertake a form of extra training but do not complete any formal speciality training. The lower agreement scores are likely due to the nature of the DWSI's work in orthodontics and not needing to routinely score IOTN as part of their clinical practice. It has been shown that dental registrants without speciality training achieve lower agreement in IOTN scoring than those who had completed orthodontic speciality training.^16^

The Easy IOTN app is designed to be a chairside reference tool, allowing clinicians to directly and quickly assess the treatment need of patients via a clinical assessment. In this investigation, plaster study models were used to determine the reliability and accuracy of the app and hence, the reported results should be viewed in this context. However, both IOTN DHC and AC scores derived from study models have been shown to provide an accurate representation of clinical observations.^21^

The majority of malocclusions scored an IOTN DHC of 4 or 5 and an AC between 4 and 9, which is reflective of malocclusions routinely accepted and treated within a secondary care orthodontic department.^22^ In order to increase the generalisability of results and to facilitate the ability to discriminate between cases eligible to be treated in the NHS, models representing milder malocclusions not qualifying for NHS treatment were included. The Easy IOTN app is designed for use by all dental clinicians of varying experience and hence does not rely on the clinician having an extensive knowledge of IOTN. Importantly, it also does not require clinicians undertaking formal IOTN calibration. Although the number of clinicians involved in this assessment could be viewed as a limitation, the generalisability of the current results is enhanced as the six clinicians assigned to score the models using the app had no formal calibration experience in the use of the conventional method and two clinicians (DWSI and OT) infrequently used IOTN in their daily practice. However, as the app has been designed to be used by all members of the dental team, future investigations should include a sample of GDPs so their reliability with using the app to assess orthodontic treatment need can be assessed. Sources of potential bias were accounted for by adhering to an agreed protocol for scoring the study models when using the app and ensuring each clinician using the app undertook training by answering the 30 questions listed in the continuing professional development section. Furthermore, selection bias was minimised as the selection of the study models was undertaken by a member of staff not involved in the scoring of the models.

## Conclusions

Within the limitations of this investigation, the Easy IOTN app was shown to have moderate inter-rater reliability. Variation in the intra-rater reliability was evident between clinicians of different grades and levels of experience. Importantly, the diagnostic accuracy of the app to discriminate between malocclusions that qualify for NHS treatment was rated at excellent (IOTN DHC) and acceptable (IOTN AC) and independent of the clinicians' grade or level of experience.
